# Identifying improvements to complex pathways: evidence synthesis and stakeholder engagement in infant congenital heart disease

**DOI:** 10.1136/bmjopen-2015-010363

**Published:** 2016-06-06

**Authors:** Sonya Crowe, Rachel Knowles, Jo Wray, Jenifer Tregay, Deborah A Ridout, Martin Utley, Rodney Franklin, Catherine L Bull, Katherine L Brown

**Affiliations:** 1 Clinical Operational Research Unit, University College London, London, UK; 2 Population, Policy and Practice Programme, UCL Institute of Child Health, London, UK; 3 Great Ormond Street Hospital NHS Foundation Trust, London, UK; 4 Royal Brompton and Harefield NHS Foundation Trust, London, UK

**Keywords:** STATISTICS & RESEARCH METHODS

## Abstract

**Objectives:**

Many infants die in the year following discharge from hospital after surgical or catheter intervention for congenital heart disease (3–5% of discharged infants). There is considerable variability in the provision of care and support in this period, and some families experience barriers to care. We aimed to identify ways to improve discharge and postdischarge care for this patient group.

**Design:**

A systematic evidence synthesis aligned with a process of eliciting the perspectives of families and professionals from community, primary, secondary and tertiary care.

**Setting:**

UK.

**Results:**

A set of evidence-informed recommendations for improving the discharge and postdischarge care of infants following intervention for congenital heart disease was produced. These address known challenges with current care processes and, recognising current resource constraints, are targeted at patient groups based on the number of patients affected and the level and nature of their risk of adverse 1-year outcome. The recommendations include: structured discharge documentation, discharging certain high-risk patients via their local hospital, enhanced surveillance for patients with certain (high-risk) cardiac diagnoses and an early warning tool for parents and community health professionals.

**Conclusions:**

Our recommendations set out a comprehensive, system-wide approach for improving discharge and postdischarge services. This approach could be used to address challenges in delivering care for other patient populations that can fall through gaps between sectors and organisations.

**Strengths and limitations of this study**

- A transparent process for incorporating a wide range of evidence was used to develop recommendations for improving multiple aspects of service delivery.
- Qualitative evidence was useful in specifying the problems in service delivery and how they might be improved, while quantitative evidence based on national audit data sets enabled prioritisation of patient groups according to their risk.
- Families and professionals from across the entire patient pathway contributed to the development of the recommendations.
- Although evidence-informed, the recommendations have not been validated and it will be important to evaluate their future implementation.

## Introduction

Outcomes following interventions for congenital heart disease (CHD) have received considerable attention within the UK since the 1990s.^1–5^ Advances in surgical interventions and hospital-based care of infants undergoing intervention for CHD have contributed to significant improvements in their short-term outcomes. This is despite a rise in the number and complexity of cases.^6^ As a consequence, the number and complexity of survivors requiring care in the community following discharge from the specialist surgical centres is increasing. To date, research has largely focused on in-hospital and short-term outcomes.^7–10^ However, there is now an established body of evidence showing appreciable mortality postdischarge (3–5%)^11^ and problems in accessing and providing appropriate support for babies and their families following discharge.^12–16^

Aligning care for patients with complex needs across organisations can be challenging, particularly when these involve rare conditions. Many initiatives are aiming to integrate care and address fractures in systems that allow individuals to ‘fall through the gaps’.^17–21^ The case for improving the alignment and coordination of services delivered across different organisations and sectors is clear. However, attempts to do so can risk exacerbating the situation through unintended negative consequences or by diverting attention and scarce resources from more effective approaches.^22–24^ Decisions about what needs to change, and how, in order to improve a service that crosses sectors are multifaceted. UK guidelines on service delivery^25^ and the development of complex interventions^26–28^ stress the importance of systematically assessing and synthesising relevant evidence. However, methods for robustly synthesising diverse forms of evidence in order to inform system-wide improvements to service delivery are not well established.^21^ ^29^ ^30^

In this article, we present an evidence synthesis aligned to a process of stakeholder engagement that culminated in a set of recommendations for improving the discharge and postdischarge management of infants undergoing intervention for CHD. This work builds directly on a programme of research that generated information regarding patient risk characteristics and the challenges encountered in accessing and providing services for this patient population.^31^ We propose that the systematic process developed and applied effectively in this work could have useful application in addressing service delivery improvement for other patient populations that can fall through the gaps between sectors and organisations.

## Methods

### Expert advisory group and parent involvement

An advisory group was established to review evidence relating to the discharge and postdischarge management of infants following surgical or catheter intervention for CHD. It comprised professionals from three tertiary cardiac centres, representatives from primary and secondary care, patient group representatives and academics from psychology, statistics, epidemiology and operational research (see online supplementary appendix 1). The group met on five occasions (each 2–3 hours) between March 2013 and June 2014 to consider emerging findings regarding UK service provision and outcomes in this patient population. The research presented to the advisory group included:

- A systematic review of potential risk factors for unexpected deaths and unplanned readmissions following discharge;^14^
- A systematic review of postdischarge surveillance or intervention programmes;^31^
- Analyses of national CHD and paediatric intensive care audit data sets, which identified patient groups with different risk of death or emergency readmission to intensive care in the year following discharge.^11^
- Interviews with parents and health professionals regarding their experiences at or following discharge;^12^ ^13^ ^32^
- An online discussion forum with parents regarding their experiences accessing support.^31^

10.1136/bmjopen-2015-010363.supp1 Supplementary appendix

The advisory group also considered quality improvement initiatives in other paediatric disciplines.^33^ ^34^ Their critique of evidence generated early in the process informed the gathering and exploration of evidence in the later stages. For example, an online discussion forum informed the questions asked within family interviews. Through a facilitated process in their final meeting, the group generated a list of candidate recommendations for improving services.

In July 2014, these candidate recommendations and a summary of the evidence were shared at a facilitated parent workshop. This comprised parents of infants who had been discharged following intervention for CHD and who then died or were readmitted to intensive care (7 parents representing 5 babies). Their views on the candidate recommendations were captured along with suggestions for additional ones.

### Developing a draft set of recommendations

**Figure 1:**
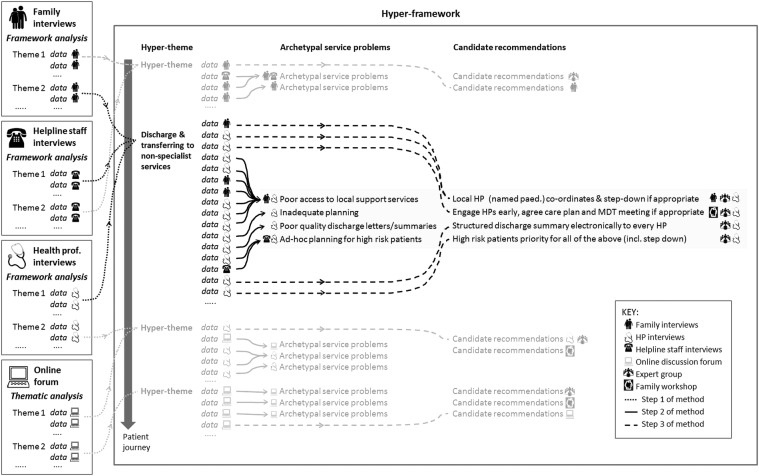
Schematic depiction of the process used to develop the draft recommendations (steps 1–3 of method). This involved creating a hyper-framework of data from four qualitative analyses (step 1, dotted arrows), identifying archetype service problems (step 2, solid arrows) and linking candidate recommendations to service problems (step 3, dashed arrows). HP, health professional; MDT, multidisciplinary team.

The research team developed and applied the following process to generate a set of draft evidence-informed recommendations. This is shown schematically in figure 1.

#### Step 1: creating a hyper-framework from four qualitative analyses

Our starting point was three separate data frameworks generated in qualitative analyses of health professional, family and helpline staff interviews,^12^ ^13^ ^31^ ^32^ and a thematic analysis of an online discussion forum.^31^ Some of the analytical themes were judged to be very similar to themes in other analyses but addressed from a different perspective (eg, a family as opposed to health professional perspective). Where this was the case they were merged into a ‘hyper-theme’ (eg, ‘discharge and transferring to non-specialist services’). A single hyper-framework was then created with each hyper-theme placed at its relevant point along a patient journey.

#### Step 2: identifying archetype service problems

**Figure 2:**
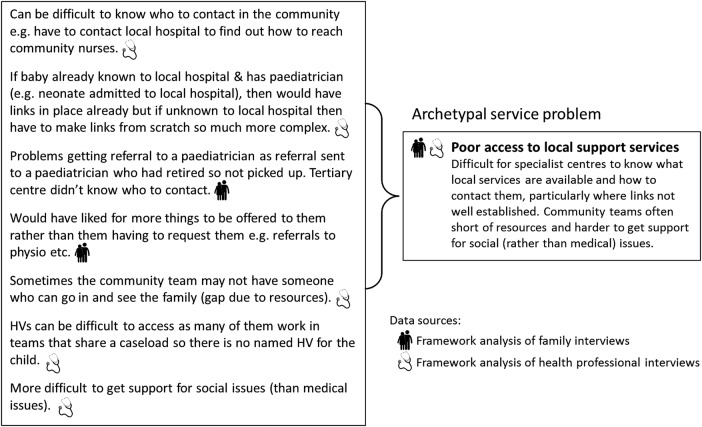
An example archetypal service problem. The data on the left-hand side were interpreted as seven different manifestations of the same archetypal problem (‘poor access to local support services’) by the research team. In this example, the data originated from two qualitative analyses: a framework analysis of family interviews^12^ ^13^ ^31^ and a framework analysis of health professional interviews.^12^ ^13^ This archetypal problem sits within the theme ‘Discharge and transferring to non-specialist centres’. HV=health visitor.

Data within hyper-themes were categorised as reflecting an underlying service problem, a candidate recommendation for improvement (eg, a cited example of good practice) or neither. Data considered neither were discarded from analysis. Within each hyper-theme, data judged to be representations of the same underlying service problem were grouped together to form a set of ‘archetypal service problems’. Each archetypal problem was thus associated with characterisations of that problem from different perspectives. For example, figure 2 shows the seven data attributed to archetypal problem ‘Poor access to local support services’.

#### Step 3: linking candidate recommendations to service problems

Candidate recommendations from the advisory group and from the parent workshop were added to the hyper-framework if they directly addressed one of the archetypal problems (eg, suggestions relating to prenatal care were considered to be outside the remit of this work). Candidate recommendations judged to be very similar to each other were then combined in order to create a set of draft recommendations linked to the archetypal problems.

### Establishing the final set of recommendations

A working group was convened to assess the draft recommendations and propose a final set for wider endorsement. This comprised selected members of the advisory group and additional representatives from the community and charitable sector (see online supplementary appendix 1 for membership). A facilitated all-day workshop was held in September 2014 (audio recorded and with live minutes), in which the group was tasked with:

- Reviewing the draft recommendations to assess the feasibility and acceptability of each;
- Assessing the draft recommendations as a set to determine priorities and targeting of recommendations to different patient groups;
- Agreeing a final set of recommendations to circulate among the full advisory group for comments and endorsement.

**Figure 3:**
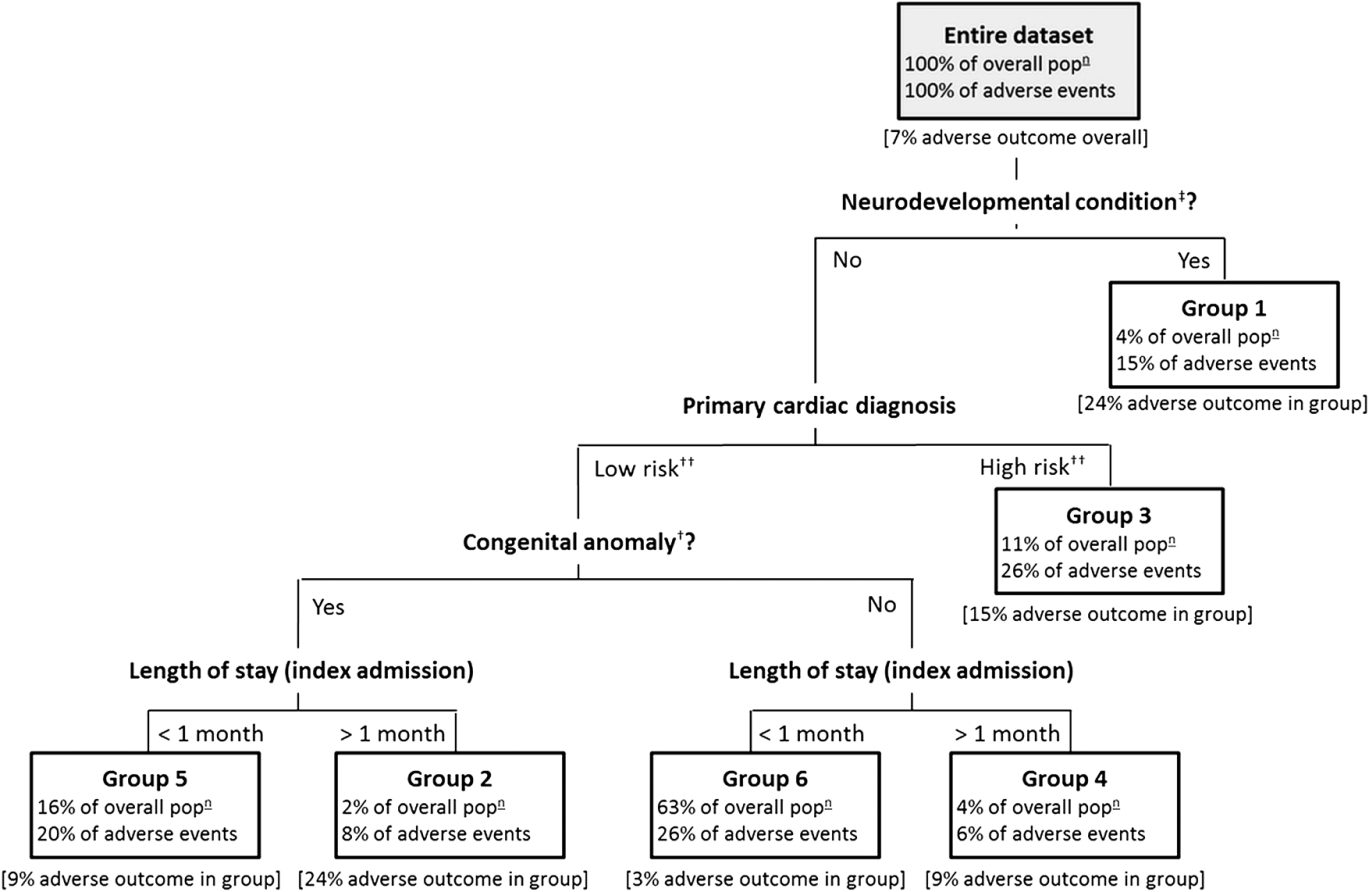
Patient-risk groups. Patient groups identified and validated in statistical analysis of national cardiac and Paediatric Intensive Care Unit (PICU) audit data.^11^ The six mutually exclusive groups are defined with respect to the following patient characteristics: absence/presence of neurodevelopmental condition‡, absence/presence of congenital anomaly†, low-risk/high-risk primary cardiac diagnosis†† and length of stay > or <1 month. Neurodevelopmental conditions and congenital anomalies were placed in groups based on the Read codes present in the audit data.^11^ For each group, we present the percentage of the overall patient population within the group, the percentage of overall adverse events* accounted for by the group and the occurrence of adverse event* in the group. *Adverse event=death (occurring outside a planned readmission) or emergency readmission to PICU within the first year postdischarge from infant cardiac surgery. ‡Neurodevelopmental conditions=a range of conditions that are likely to have lifelong impact, for example, epilepsy/seizures, developmental delay, sleep apnoea, hydrocephalus, retinopathy of prematurity, stroke, hemiparesis/hemiplegia, anoxic encephalopathy, cerebral venous sinus thrombosis and cerebral palsy. †Congenital anomalies=a range of major anomalies (some requiring neonatal surgery) with an impact that is likely to be lifelong, for example, Down syndrome, 22q11 deletion (Di George) syndrome, urogenital/ renal malformations, tracheal/trachea-oesophageal malformations, vision/hearing deficits and exomphalos/gastrointestinal malformations. ††High-risk primary cardiac diagnosis=hypoplastic left heart syndrome, functionally univentricular heart or pulmonary atresia with intact ventricular septum. Low-risk primary cardiac diagnosis=all other cardiac diagnoses.

To determine priorities and the targeting of interventions, the working group explicitly considered the level and nature of the risk associated with each of several patient groups identified from analysis of national audit data (figure 3).^11^ The groups are defined by combinations of risk factors known at the point of discharge and differ in terms of risk of death or emergency readmission to paediatric intensive care within a year of discharge. Risk factors include: presence of a neurodevelopmental condition such as cerebral palsy (see ‡ in figure 3), a congenital anomaly such as urogenital/renal malformations (see † in figure 3) and a ‘high-risk’ primary cardiac diagnosis (complex CHD such as hypoplastic left heart syndrome or pulmonary atresia where the sole source of pulmonary blood supply after neonatal surgery is a systemic-to-pulmonary arterial shunt; see †† in figure 3). Given the limited resources available to these services and recognising the different levels of resource associated with proposed improvements, the working group agreed to consider targeting recommendations to patient groups on the basis of their profiles of risk.

## Results

**Figure 4:**
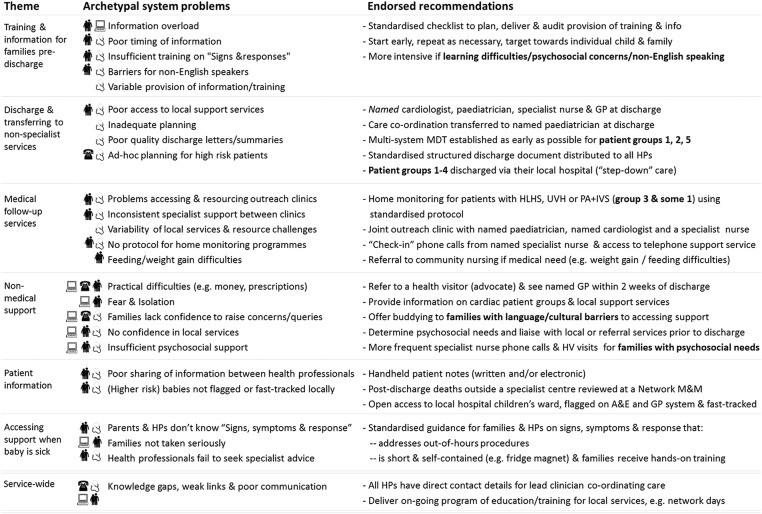
Endorsed recommendations for addressing archetype service problems. The archetype service problems generated from evidence (left-hand side) are grouped in themes with linked recommendations for service improvements (right-hand side). Data originated from qualitative analyses of family interviews 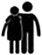,^12^ ^13^ ^31^ ^32^ health professional interviews 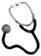,^12^ ^13^ ^31^ ^32^ helpline staff interviews 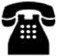 31 and an online discussion forum 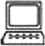.^31^ A&E, accident and emergency department; GP, general practitioner; HLHS, hypoplastic left heart syndrome; HP, health professional; IVS, intact ventricular septum; MDT, multidisciplinary team; M&M, mortality and morbidity meeting; PA, pulmonary atresia; UVH, functionally univentricular heart.

Figure 4 shows the archetypal service problems located along the patient journey from preparation for discharge through to accessing support in the community when problems arise (see online supplementary appendix 1 for additional details). The original sources of evidence contributing to each archetype are noted. The final set of service recommendations endorsed by the advisory group is set out in full in the online supplementary appendix 1 and summarised schematically in figure 4 alongside the service problems they address. We note that these include recommendations for further research. The main recommendations are presented in box 1.

Box 1 **Headline recommendations. The headline endorsed recommendations for improving services for infants who have undergone intervention for congenital heart disease**

Structured discharge and transfer of care:

- At discharge from the specialist centre, all patients should have a named cardiologist, named paediatrician (with expertise in cardiology where possible) and named specialist nurse (eg, cardiac liaison role or equivalent). Where it is not possible to allocate a named specialist nurse, there should be a named specialist nursing team. Responsibility for ensuring this lies with the specialist centre.
- At discharge home (either from the specialist centre or from local hospital if step-down), all patients should also have a named general practitioner and a named pharmacist (if discharged with a long-term prescription).
- All patients should have a nationally standardised structured discharge document that is distributed electronically to all of the health professionals involved in their care.
- Patient groups 1–4 should receive ‘step-down’ care, that is, discharge via their local hospital. Ideally this should be as an in-patient (even if just for 24 h). If this is infeasible due to bed shortages, then they should be admitted as a day case. At a minimum (given severe resource constraints), they should be seen as an outpatient as soon as possible (eg, within 48 hours).

Home monitoring for patients with hypoplastic left heart syndrome (HLHS), functionally univentricular heart or pulmonary atresia:

- Home monitoring should be provided for all patients with a primary diagnosis of HLHS, functionally univentricular heart or pulmonary atresia with intact ventricular septum. This will include all patients in group 3 and some in group 1. There should be a nationally agreed protocol for home monitoring of these patients that is based on the best available evidence. The expert group recommends that further research is conducted on the effectiveness of constituent components of home monitoring.

Guidance on signs, symptoms and response (eg, an early warning tool):

- All families and all of the health professionals involved in their support should receive the same clear guidance on ‘what is normal’ for that child, signs and symptoms to look for, how to respond and important contact numbers, for example, in the form of an early warning tool. Ideally the format and content of this guidance should be standardised nationally, with scope for tailoring to local areas/networks as appropriate.
- The expert group agreed that there is an urgent need for such guidance (eg, early warning tool) to be developed, that it should be evidence-based as far as possible and that its implementation should be evaluated (ie, its impact on families and health professionals monitored).

Information and training for families prior to discharge:

- Health professionals should use a nationally standardised checklist in order to plan, deliver and audit the provision of training and information for all families prior to discharge.

Network review of deaths outside specialist centre:

- The postdischarge death of any patient outside a specialist centre should be reported to the specialist centre and reviewed for quality improvement purposes at a morbidity and mortality meeting held by the linked network of healthcare providers.

Family buddying:

- All families should be offered an opportunity to connect with other families (eg, through social media or charity support groups) and those families more likely to experience language/cultural barriers to accessing support should be offered buddying. The expert group notes that there would need to be appropriate infrastructure to support this (eg, training for buddies) and that it may be best facilitated through the charity sector.

Many recommendations apply to all infants as they require few resources and/or were considered to be important for everyone (eg, a structured discharge document). However, some target certain patient profiles (eg, multidisciplinary care teams for children with long-term complex needs in addition to their primary cardiac diagnosis). Given that over half of all adverse events occur in the 21% of patients in risk groups 1–4 (see figure 3), recommendations regarding costly interventions were prioritised for these high-risk patient groups (eg, discharge home via the local hospital). Finally, on the basis of qualitative evidence suggesting that support can be harder to access for families with learning difficulties or for those facing cultural or language barriers,^12^ ^13^ some recommendations were prioritised for these groups (eg, offering family buddying).

## Discussion

### Principal findings

We have developed evidence-informed recommendations for managing the discharge and postdischarge care of infants following major cardiac intervention. These address directly the known challenges in this previously neglected section of the care pathway. Importantly, within the context of resource constrained services, these recommendations incorporate the targeting of some interventions at certain patient groups on the basis of the level and nature of their risk, as well as the relative size of the group. Our work involved developing and applying a process for systematically synthesising evidence accrued from diverse sources (including published literature, quantitative and qualitative analyses), and feeding this into a structured multistakeholder decision-making process. This process could be used to address challenges in delivering ongoing care for other patient populations who require multiple services.

### Findings in relation to other studies

Many other fields of medicine have reduced deficits in communication and information transfer at hospital discharge using standardised and structured handover documentation:^35–42^ these could usefully inform the codevelopment, piloting and evaluation of one for infant CHD. Similarly, a checklist to support training for families prior to discharge should be informed by similar initiatives elsewhere, for example, in neonatal intensive care.^43–45^ Our recommendation that such a checklist addresses the needs of non-English-speaking families or parents with psychosocial or learning difficulties is in line with findings regarding vulnerable patients more generally.^46^ Our recommendation of an early warning tool for use with parents and community health professionals involved in the care of infants with CHD is supported by evidence of early warning tools being used to detect signs of deterioration in children presenting to accident and emergency by non-specialist practitioners and^47^ evidence that the timely recognition of a deteriorating child using standardised criteria leads to a greater chance of rescue in the context of paediatric infections such as meningococcal disease.^48^ ^49^

### Strengths and weaknesses

Deciding how best to deliver effective and efficient services across sectors is challenging and inevitably involves an element of subjectivity, not least because there is often limited or disparate evidence that is difficult to synthesise. In this context, our study had two key strengths. First, developing and applying a systematic and transparent process for synthesising and incorporating a broad range of available evidence covering multiple aspects of the problem enhanced the richness and breadth of the recommendations. For example, the qualitative evidence was very useful in specifying what the problems in services were and how they might be addressed, while the quantitative evidence enabled prioritisation. Second, representatives from across the entire patient pathway critiqued the feasibility and acceptability of the recommendations, and the needs of service users remained of central importance through incorporating findings from an online discussion forum and interviews, views captured in a family workshop and involving a parent representative on the expert group. Although evidence-informed, the recommendations have not been validated and it will be important to evaluate their future implementation.

### Implications for policymakers and clinicians

The recommendations are of direct relevance to all healthcare professionals caring for infants with CHD including general practitioners, community nurses, health visitors, secondary care paediatricians and clinicians in specialist surgical centres. They are also of importance to patients, their families and support groups. CHD services in the UK are currently under national review and the recommendations reported in this article fed into the Review's public consultation on care standards and service specifications to be used in commissioning specialist CHD services.^50^

The recommendations of this study will have resonance in other settings where such postdischarge deaths have been reported, including Germany^51^ and the USA.^52^ ^53^ There is evidence of poor transfers and continuity of care across sectors in many other patient populations with needs that require a range of health services,^54^ in particular other complex patient groups where postdischarge care pathways are associated with the loss of vulnerable patients to follow-up,^55^ late unexpected deaths^56^ and undetected pathology.^57^ Our study provides an exemplar of how to approach such problems systematically, which is becoming increasingly important within the context of policy ambitions to move care closer to home. Our approach may be relevant in the development of complex interventions^26–28^ and service guidelines more broadly.^26^

### Unanswered questions and future research

Evidence suggests that there are cultural and language barriers to accessing support following intervention for infant CHD and that health outcomes differ between ethnic groups.^11^ ^32^ However, insufficient data were available to understand fully the reasons for these differences and so further research on health inequalities is a priority. Despite a number of studies reporting efficacy of home monitoring programmes,^51^ ^52^ ^58–63^ further research is needed to ascertain the efficacy of individual constituent components (eg, frequency of oxygen saturation and/or weight monitoring, feeding intervention, use of breaching criteria), which vary across programmes. Finally, we note that future work developing, piloting and evaluating standardised home monitoring, training and early warning tools should include consideration of how to collect, analyse and use outcome and process measures appropriately, which could be informed by process measures developed for assessing transitional care of other vulnerable patients.^38^ ^39^ ^64^
