# Supplementary material for: Identifying improvements to complex pathways: evidence synthesis and stakeholder engagement in infant congenital heart disease
Source: BMJ Open. 2016 Jun 6;6(6):e010363. doi: 10.1136/bmjopen-2015-010363 (PMC4908909; doi:10.1136/bmjopen-2015-010363)
Supplement: Supplementary appendix [file bmjopen-2015-010363supp.pdf]

Online supplementary appendix 1: Recommendations for service improvements

The table below sets out the service challenges identified from evidence [1-6] along with recommendations for service improvements endorsed by the expert advisory group and working group. It is reproduced with permission from reference [1].

Expert advisory group

- Nick Barnes, Consultant paediatrician with expertise in cardiology (Northampton General Hospital)
- David Barron, Consultant cardiac surgeon (Birmingham Children's Hospital)
- Kate Brown, Consultant paediatric cardiac intensivist and Principal investigator (Great Ormond Street Hospital for Children)
- Kate Bull, Senior lecturer in cardiology and medical advisor on family policy (Great Ormond Street Hospital for Children)
- Sonya Crowe, Senior research associate in operational research (University College London)
- Piers Daubeney, Consultant paediatric and fetal cardiologist (Royal Brompton Hospital)
- Kay Dyer, Cardiac liaison nurse (Birmingham Children's Hospital)
- Christie Fox, Children's cardiac nurse specialist (Royal Brompton Hospital)
- Rodney Franklin, Consultant paediatric cardiologist (Royal Brompton Hospital)
- Sally Hull, General practitioner and reader in primary care (Jubilee Street Practice, NHS East London and the City)
- Samantha Johnson (Children's Heart Federation)
- Rachel Knowles, Clinical research fellow (paediatric epidemiology) (University College London)
- Deborah Ridout, Senior research fellow in statistics (University College London)
- Samana Schwank (Children's Heart Federation)
- Helen Silk, former Cardiac liaison nurse at Great Ormond Street Hospital for Children (now at Brighton and Sussex University Hospitals)
- Liz Smith, Advanced nurse practitioner (Great Ormond Street Hospital for Children)
- Jeni Tregay, Clinical psychologist (Great Ormond Street Hospital for Children)
- Jo Wray, Health psychologist and senior research fellow (Great Ormond Street Hospital for Children)

Working group

- Nick Barnes, Consultant paediatrician with expertise in cardiology (Northampton General Hospital)
- Kate Bull, Senior lecturer in cardiology and medical advisor on family policy (Great Ormond Street Hospital for Children)
- Hannah Charrot, Community children's nurse (Cambridgeshire Community Services)
- Rodney Franklin, Consultant paediatric cardiologist (Royal Brompton Hospital)
- Sally Hull, General practitioner and reader in primary care (Jubilee Street Practice, NHS East London and the City)
- Suzie Hutchinson, Parent/patient representative (Little Hearts Matter)
- Jan Pennington, Health visitor (Barts Health NHS Trust)
- Liz Smith, Lead advanced nurse practitioner (Great Ormond Street Hospital for Children)

References

[1] Brown KL, Wray J, Knowles RL, et al. Infant deaths in the UK community following successful cardiac surgery: building the evidence base for optimal surveillance, a mixed-methods study. *Health Serv Deliv Res* 2016;4(19).

[2] Crowe S, Ridout DA, Knowles R, Tregay J, Wray J, Barron D, et al. Death and emergency readmission of infants discharged after interventions for congenital heart disease: A national study of 7643 infants to inform service improvement. *J Am Heart Assoc* 2016;5:e003369.

[3] Tregay J, Wray J, Bull C, Franklin RC, Daubeney P, Barron DJ, et al. Unexpected deaths and unplanned re-admissions in infants discharged home after cardiac surgery: a systematic review of potential risk factors. *Cardiol Young* 2014;1–14.

[4] Tregay J, Wray J, Crowe S, Knowles R, Daubeney P, Franklin R, et al. Going home after infant cardiac surgery: A qualitative study. *Arch Dis Child* 2016;101:320–5.

[5] Tregay J, Brown K, Crowe S, Bull C, Knowles R, Smith L, et al. Signs of deterioration in infants discharged home following congenital heart surgery in the first year of life: A qualitative study. *Arch Dis Child* 2016; doi: 10.1136/archdischild-2014-308092.

[6] Tregay J, Brown K, Crowe S, Bull C, Knowles R, Wray J. "I was so worried about every drop of milk" – feeding problems at home are a significant concern for parents after major heart surgery in infancy. *Matern Child Nutr* 2016;1740–8709.

**Identified service challenges and barriers to support [Source]**<br>[FI] - family interviews ; [HPI] - health professional interviews;<br>[HLI] - helpline staff interviews; [OF] - online user forum.	**Recommendations and comments**
**<u>1. Training & information for families pre-discharge</u>**<br><br>**Information overload:** Lots of information, some of which families find difficult to understand and absorb. [FI, OF]<br><br>**Poor timing:** Information is often rushed before discharge. [HPI, FI]<br><br>**Insufficient training on "Signs, symptoms, responses":** Often missed, vague or unstructured, and no written material to take away. [HPI, FI]<br><br>**Barriers for non-English speakers:** Limited funding and/or access to interpreters and most resources only available in English. [HPI, FI]<br><br>**Some families miss out:** "Hit & miss" which families are offered what information & training, depending on the health professional at bedside and resource shortages. [HPI]	1.1. **For all patients**, training and information should start as early as possible, repeating as necessary and checking that families have taken it on board.<br>1.2. **For all patients**, information should be targeted towards the individual child, e.g. through hands-on demonstrations with their baby and involvement in the baby's care whilst in hospital. Nationally standardised generic information should also be provided, e.g. in written form and as a web-based resource.<br>1.3. **For all patients**, training and information should be provided as far as possible in the format most helpful for the family and should therefore be available in a range of formats (e.g. verbal, written, visual, and digital). Video and other visual information was considered easier to understand, particularly for non-English speakers. E.g., mobile phone videos recorded at discharge of the 'normal' status of the child could be used by families as a comparison when child is unwell.<br>1.4. **For all patients**, health professionals should use a nationally standardised checklist in order to plan, deliver and audit the provision of training and information for families prior to discharge. The expert advisory group will propose a checklist (based on the evidence gathered in the Study), which they recommend should be piloted and evaluated.<br>1.5. The expert advisory group notes that the following patient/family groups would benefit from more frequent/intensive provision of recommendations 1.1-1.4:<br>• **Non-English speaking families**<br>• **Families with learning difficulties**<br>• **Families with psychosocial concerns**<br>1.6. **For these patient/family groups**, the expert advisory group also recommends referral to a health visitor/social work team to assist in ongoing training support (in their own home).<br>1.7. **For non-English speakers**, the expert advisory group recommends interpreters and translations of written material where this is feasible and appropriate for the family but notes that where this is not possible visual information (e.g. videos of their own child) should be used.
**<u>2. Discharge & transferring to non-specialist services</u>**<br><br>**Poor access to local support services:** Difficult for specialist centres to know what local and community services are available and how to contact them (e.g. named individuals rather than teams), particularly where links not well established. Community teams are often short of resources and it's harder to get support for social (rather than medical) issues. [HPI, FI]	2.1. At discharge from the specialist centre, **all patients** should have a named cardiologist, named paediatrician (with expertise in cardiology where possible) and named specialist nurse (e.g. cardiac liaison role or equivalent). Where it is not possible to allocate a named specialist nurse, there should be a named specialist nursing team. Responsibility for ensuring this lies with the specialist centre.<br>2.2. At discharge home, either from the specialist centre or from the local hospital if step-down, **all patients** should also have a named GP and a named pharmacy (if discharged with a long-term prescription).<br>2.3. For **all patients**, responsibility for care co-ordination should be transferred to the named paediatrician at discharge from the

**Inadequate planning:** Can be ad-hoc and strongly influenced by the availability and accessibility of local resources, leading to variation across the country in terms of who is offered what follow-up care. Contact with local services is often made on the day of discharge, at which point it is difficult to organise appropriate timely support. [HPI]<br><br>**Poor quality discharge letters/summaries:** Often very delayed, don't go to all health professionals, contain too much specialist information and terminology and often don't include: basic information (e.g. wound care, immunisations); what training families have received; details on what needs to be monitored and any associated breach criteria; what to look out for and how to respond. [HPI]<br><br>**Ad-hoc planning for high risk patients:** Often no protocol in place for identifying high risk babies and the (extra) care that is offered to them - large variability across the country. [HPI, HLI]	specialist centre. The named paediatrician and GP are responsible for referring to local services and maintaining effective communication between health professionals.<br>2.4. A multidisciplinary team should be established as early as possible (2-3 days prior to discharge or earlier) for the following groups:<br>• **Patient groups 1, 2, 5** (and **4** where appropriate) [multi-system]<br>• **Families with psychosocial issues** (across **all patient groups**) [psychosocial involvement]<br>This should involve phoning the health visitor and community nursing teams prior to discharge and, where appropriate, inviting them to discharge planning meetings.<br>2.5. **All patients** should have a nationally standardised structured discharge document that is distributed electronically to all relevant health professionals. The expert advisory group will propose the minimum content for this discharge document (based on the evidence gathered in the Study), which they recommend should be piloted and evaluated.<br>2.6. For **all patients**, the structured discharge document should be used for:<br>• Developing a child-specific care plan<br>• Discharge planning with the family, with discussions starting as early as possible (at admission).<br>2.7. Weekend discharge without appropriate support or sharing of information should be avoided **for all patients**.<br>2.8. **Patient groups 1-4** should receive "step-down" care, i.e. discharge via their local hospital. Ideally this should be as an in-patient (even if just for 24 hours). If this is infeasible due to bed space they should be admitted as a day case. At a minimum (given resource constraints) they should be seen as an outpatient as soon as possible (e.g. within 48 hours).
<u>**3. Medical follow-up services**</u><br><br>**Problems with clinics:** Clinics are often full and running late. Specialist centre outpatient clinics can be difficult for families to get to and are not always attended by specialist nurses. Not all outreach clinics are jointly run with paediatrician or attended by specialist nurses and, in general, there is no multi-professional follow-up. [HPI, FI]<br><br>**Inconsistent specialist support between clinics:** Many families (particularly "high risk") get regular calls from CLNs/CNSs, but some don't and can find it hard to get in touch with them. Families often don't speak with the same nurse each time. [HPI, FI]<br><br>**Variability & resource challenges:** The use of local services is not standardised, the support available varies across the country and there are often insufficient resources. In particular, there are not enough paediatricians with expertise in cardiology (or out-of hours/annual leave cover) and often newly trained or less experienced community nurses / health visitors attend visits (sometimes from a pooled resource so lack consistency of care). Babies must have a medical need to get a community nurse but it can be difficult to maintain regular home visits from health visitor as the baby may not be considered high priority (i.e. child protection). [HPI]	3.1. **All patients** should be seen by their named paediatrician and named cardiologist at joint outreach clinics. A specialist nurse should attend all outpatient clinics and outreach clinics.<br>3.2. **All families** should receive "check-in" telephone calls from their named specialist nurse (team), the frequency of which should be determined by their needs.<br>3.3. **All families** should have access to a telephone support service led by specialist nurses.<br><br>The expert advisory group notes that recommendations 3.1-3.3 are broadly in line with the proposed standards from the NHS England Review. They further note that in order to meet these recommendations additional resources may be required in some areas.<br><br>3.4. **All patients with a medical need** (such as weight gain / feeding difficulties) should have access to community nursing and **all patients** should be referred to a health visitor team (via the GP or through community child health services). It is important that the community nurses / health visitors are supported by the specialist centre (see 7.1-7.3).<br>3.5. Home monitoring should be provided for **all patients with a primary diagnosis of hypopastic left heart syndrome, functionally univentricular heart or pulmonary atresia with intact ventricular septum**. This will include **all patients in group 3 and some in group 1**.<br>3.6. There should be a nationally agreed protocol for home monitoring of these patients, based on the best available evidence. The expert advisory group recommends that further research is conducted on the effectiveness of constituent components of home monitoring. The expert advisory group notes that in order to meet the needs of a larger number of home monitoring patients,

**No protocol for home monitoring:** Large variation between centres in the provision of home monitoring programmes (which babies and what it consists of) and generally no clear protocol. Often a lack of clarity amongst community nurses / health visitors / families as to what to do with the information monitored. Some families find it helpful, others a distraction or too complicated. [HPI, FI]<br><br>**Feeding/weight gain:** Very stressful aspect of care for many families, who often feel unsupported and receive conflicting advice from the specialist centre, local hospital and health visitor. Replacing nasogastric tubes out of hours is particularly stressful. [FI]	community nurses may need to run clinics or skype clinics rather than provide home visits.<br>3.7. The structured discharge document that is shared electronically with all health professionals should contain:<br>• The home monitoring protocol (with criteria specific to the individual child) **(patient group 3** and, where appropriate, **1)**<br>• Clear guidelines on feeding requirements, including what is normal and expected for the individual child **(patients with weight gain / feeding difficulties)**.<br>.
**<u>4. Non-medical support</u>**<br><br>**Practical difficulties:** Families sometimes experience practical difficulties in the community that may not have been identified prior to discharge. These include: child care for siblings, access to transport to get to follow-up appointments, financial difficulties due to long hospital stays, debts, loss of earnings and inability to return to work. Some families struggle to adhere to medication regimes and can experience difficultly getting prescriptions because GPs are not always clear what has been prescribed or what to do about off-license medications. [OF, HLI, FI]<br><br>**Fear & isolation:** Parents often live in fear of an emergency and the worry of infection isolates them from other parents and support groups in their community. [OF, FI]<br><br>**Families lack confidence:** Some families lack the confidence to approach or challenge health professionals, fail to ask questions during appointments for fear of appearing ignorant or incapable, or lack the ability to articulate their concerns (particularly non-English speakers). [OF, HLI, HPI]<br><br>**The strain of "expert parenting**" / **lack of confidence in local services:** Many families have to explain/pass on information about their child's condition to health professionals that don't have specialist knowledge and sometimes (as the holders of knowledge) feel they are battling with local services. Many families take on an "expert parent" role, which can be alienating and frightening. [OF, FI, HPI]<br><br>**Insufficient psychosocial support:** Support offered to families is often	4.1. **All families** should have a named GP and named pharmacy prior to discharge. Changes to medication in hospital or as an outpatient should be sent electronically (email/fax) to the named GP and pharmacy within an agreed timeframe (e.g. 72 hours).<br>4.2. **Families with non-medical needs** should be guided by their local health professionals (e.g. GP, health visitor, community nurse) towards local support services appropriate to their needs (e.g. charity support or a family support worker). The expert advisory group notes that statutory services for non-medical support are limited and declining and that this role is increasingly met by non-statutory services. The expert advisory group recommends that further information is established regarding:<br>• The range of support services available from non-statutory groups and how health professionals can keep up to date with this (e.g. establishing a Directory that is available to families and health professionals)<br>• The proportion (and range) of families that currently seek support from such services and their experiences in doing so<br>4.3. **All families** should be offered an opportunity to connect with other families (e.g. through social media and charity support groups) and **those families more likely to experience language/cultural barriers** to accessing support should be offered buddying. The expert advisory group noted that there would need to be appropriate infrastructure to support this (e.g. training for buddies) and that this may be best facilitated through the charity sector. Ideally, **families with learning difficulties** or **non-English speaking families** should be offered more intensive help and encouragement to access support networks and **group 3 patients** should be offered buddying with other group 3 patients specifically.<br>4.4. **All patients** should be referred to a health visitor (via the GP or through community child health services), who can act as an advocate for them, e.g. in helping them to articulate concerns / questions at appointments.<br>4.5. **All patients** should be provided with information regarding patient support groups, both by the specialist centre (in particular cardiac support groups) and local health professionals (local support services that may be more generic such as child development clinics).<br>4.6. **For all patients**, the named GP and named paediatrician should act as consistent points of contact in their locality.<br>4.7. As part of the discharge planning process, families' expectations of local/community health services should be managed, with relationships established as early as possible. Specifically, **all patients** should see their named GP within 2 weeks of discharge.<br>4.8. Psychosocial meetings should be held after ward rounds in the specialist centre (led by the lead specialist nurse and psychologist)

purely related to the medical needs of their child with no specific protocol for assessing their psychosocial needs and resources harder to get for social (rather than medical) support unless they meet criteria for safeguarding. [OF, HPI, FI]	in order to determine needs and liaise with local or referral services as appropriate.<br>4.9. **Families with psychosocial needs** should receive more frequent phone calls ("checking in") from their named specialist nurse (team) and additional visits from a health visitor who is able to provide support and refer on to a psychologist if necessary.
**5. Patient information**<br><br>**Poor sharing of patient information:** There are very few shared electronic patient record systems across services and notes from clinics/visits are often not sent to the other health professionals involved (often assuming that they will be forwarded on by someone else, e.g. GP). Information is often relayed through the families, although there is inconsistency in the extent to which health professionals use Red Books, hand-held records, health booklets etc. [HPI,FI]<br><br>**Not flagged or fast-tracked:** Often no formal system for flagging (high risk) babies to local health professionals or for enabling them to have quick access to services. [HPI, FI]	5.1. **For all patients**, every health professional involved in their care should receive electronic versions of:<br>• The structured discharge document (see 2.5).<br>• Patient-specific information about signs, symptoms and guidance on what to do (see 6.1).<br>5.2. **All families** should have handheld notes (written and/or electronic). The format and content of these should ideally follow a national standard and is likely to include the structured discharge document (see 2.5).<br>5.3. **For all families**, their clinic letters should be sent electronically to the entire multidisciplinary team within a nationally agree standard timeframe (e.g. 72 hours).<br>5.4. The post-discharge death of **any patient** outside a specialist centre should be reported to the specialist centre and reviewed for quality improvement purposes at a morbidity and mortality meeting held by the linked network of healthcare providers.<br>5.5. **All patients** should have open access to their local hospital children's ward.<br>5.6. **All patients** should be flagged on their local hospital A&E system (e.g. using flags on the Patient Administration System), with fast-track referral to a secondary care paediatrician.<br>5.7. **All patients** should be flagged on their GP practice system with clear instructions for receptionists / other GPs regarding appropriate fast-tracking.
**6. Accessing support when a baby is sick**<br><br>**Not knowing "Signs, symptoms, response":** Parents and local health professionals are often unclear what signs & symptoms to look for (or threshold criteria) and how to respond, with insufficient guidance from specialist centres. [HPI,FI]<br><br>**Families not taken seriously:** Families sometimes find it difficult to verbalise their concerns, lack the confidence to seek help or don't feel listened to by health professionals when they do. [OF, FI]<br><br>**Failing to seek specialist advice:** Sometimes local health professionals fail to notify the paediatrician with expertise in cardiology or specialist	6.1. **All families** and all of the health professionals involved in their support should receive the same clear guidance on "what is normal" for that child, signs & symptoms to look for, how to respond and important contact numbers, e.g. in the form of a traffic light tool. Ideally the format and content of this guidance should be standardised nationally, with scope for tailoring to local areas/networks as appropriate.<br>6.2. The expert advisory group agreed that there is an urgent need for such guidance (e.g. traffic light tool) to be developed, that it should be evidence-based as far as possible and that its implementation should be formally evaluated (i.e. its effectiveness and impact on families and health professionals monitored).<br>6.3. The expert advisory group recommends that guidance addresses out-of-hours procedures and specifies that the named specialist nurse or cardiologist must be informed if any patient attends hospital (for any reason). The expert advisory group also recommends that guidance is short and self-contained (e.g. a sheet in the Red Book, fridge magnet, credit card for wallet, phone app) and notes that it is likely to require different content for the following four sets of patients:

centre of an incident (e.g. A&E visit) or contact them when there is a concern, even when they lack the specialist knowledge they need (particularly out-of-hours). [HPI, FI]	• Group 1<br>• Groups 2 and 4<br>• Patients with a primary diagnosis of hypoplastic left heart syndrome, functionally univentricular heart or pulmonary atresia with intact ventricular septum. This includes all of Group 3 and some patients in Group 1.<br>• Groups 5 and 6<br>Each version of the guidance should have the scope for customisation to the individual needs of the patient.<br>6.4. **All families** should receive hands-on training in the guidance prior to discharge. The expert advisory group suggests that the guidance (e.g. traffic light tool) is also made available to families in a diary format as this may empower them to seek help and articulate their concerns with health professionals in a timely fashion.<br>Note that some of the recommendations in sections 4 and 7 are also relevant here.
**<u>7. Knowledge gaps, weak links & poor communication between health professionals</u>**<br><br>All strands of the qualitative evidence highlighted the existence (across the entire patient journey) of large knowledge gaps between specialist and non-specialist health professionals, weak links across different sectors and poor communication between health professionals, identifying these as major potential or actual causes of failures in care. Examples reported in the data include:<br>- Absence of anyone to coordinate across specialties<br>- Difficulty knowing who is taking responsibility for what<br>- Unclear who to contact, when<br>- Information lost between hospitals/ health professionals<br>- A reliance on families to transfer information/knowledge<br>- Hierarchical communication between health professionals<br><br>These issues were related particularly to care that did not fall within existing "networks" or well-establish personal links between professionals (that had built a rapport and trust over time). [HPI, FI, HLI, OF]<br><br>Examples of effective communication and co-ordination were also reported, typically when there was a paediatrician with expertise in cardiology running joint clinics with the cardiologist who was familiar with the specialist centre processes and had direct contact with individuals there. Often Cardiac Specialist Nurses provided the link and point of contact between local and specialist centres and this was reported to work particularly well when they attended local clinics and/or trained key link nurses in the local team. [HPI, FI]	7.1. The expert advisory group highlights the importance of strengthening networks and building local capacity in order to address the large knowledge gaps between specialist and non-specialist health professionals, weak links across different sectors and poor communication between health professionals, which were identified as major potential or actual causes of failures in care.<br>7.2. In particular, the expert advisory group recommends that all health professionals involved in caring for **any patient** should have the direct contact number and email for the lead clinician co-ordinating care, i.e. the named paediatrician, in order to ask questions or raise concerns. They should additionally have the contact number/email for the named specialist nurse and cardiologist at the specialist centre. This contact information will be contained on the discharge document (see 2.5).<br>7.3. Further suggestions include:<br>• Deliver an on-going program of education/training for local services, for example specialist centre running study days and networking days for community health professionals<br>• Increase nationally the number of paediatricians with expertise in cardiology and specialist nurses<br>• Have cardiac trained nurses in the community (or formal training once a year for community paediatric nurses)<br>• Train key link nurses in local hospitals to establish direct links and familiarity with specialist centre protocols<br>• Have a CHD repository at the local hospital as part of the paediatric team's knowledge base<br>• Have a named healthcare professional (formalised with protected time) locally, such as a Paediatrician with Expertise in Cardiology, that can liaise with the specialist centre and come to network study days

Reproduced with permission from Brown et al. Health Serv Deliv Res 2016;4(19).
